# Online Continual Learning in Acoustic Scene Classification: An Empirical Study

**DOI:** 10.3390/s23156893

**Published:** 2023-08-03

**Authors:** Donghee Ha, Mooseop Kim, Chi Yoon Jeong

**Affiliations:** 1 Artificial Intelligence Research Laboratory, Electronics and Telecommunications Research Institute, 218 Gajeong-ro, Daejeon 34129, Republic of Koreagomskim@etri.re.kr (M.K.); 2 Artificial Intelligence, University of Science and Technology, 217 Gajeong-ro, Daejeon 34113, Republic of Korea

**Keywords:** acoustic scene classification, catastrophic forgetting, continual learning, intransigence, online learning

## Abstract

Numerous deep learning methods for acoustic scene classification (ASC) have been proposed to improve the classification accuracy of sound events. However, only a few studies have focused on continual learning (CL) wherein a model continually learns to solve issues with task changes. Therefore, in this study, we systematically analyzed the performance of ten recent CL methods to provide guidelines regarding their performances. The CL methods included two regularization-based methods and eight replay-based methods. First, we defined realistic and difficult scenarios such as online class-incremental (OCI) and online domain-incremental (ODI) cases for three public sound datasets. Then, we systematically analyzed the performance of each CL method in terms of average accuracy, average forgetting, and training time. In OCI scenarios, iCaRL and SCR showed the best performance for small buffer sizes, and GDumb showed the best performance for large buffer sizes. In ODI scenarios, SCR adopting supervised contrastive learning consistently outperformed the other methods, regardless of the memory buffer size. Most replay-based methods have an almost constant training time, regardless of the memory buffer size, and their performance increases with an increase in the memory buffer size. Based on these results, we must first consider GDumb/SCR for the continual learning methods for ASC.

## 1. Introduction

Acoustic scene classification (ASC) involves classifying a sound event into a predefined acoustic scene class [1,2,3,4]. Because deep-learning-based methods significantly improve performance in various tasks, numerous deep learning methods have been applied to ASC tasks to improve the classification accuracy of sound events [5,6,7,8,9,10,11,12,13,14,15]. In the detection and classification of acoustic scenes and events (DCASE) challenge, which is a representative event in the field of ASC, deep-learning-based methods improved the performance of sound event classification [10,12]. However, if a trained model with the DCASE dataset is deployed directly in the real world, unexpected results with severe performance degradation are often obtained. This phenomenon occurs because of device heterogeneities, that is, differences between the device used to build the training dataset and the device using the trained model. In addition, depending on the user’s environment, predefined classes may not be able to identify the right class assignment when there is no exact class matching. Therefore, to deploy a network model in the real world, re-training or fine-tuning the network model with new data obtained by the user’s sensors is inevitable [7,16]. However, re-training or fine-tuning the neural network incurs considerable computational costs and a large amount of memory space for training data.

Continual learning (CL) methods attempt to resolve the problems of task changes, including those associated with device heterogeneity, and class-incremental and task-incremental scenarios by transferring the knowledge obtained from previous tasks to the new classification task. Several studies on CL have been conducted, and various mechanisms have been proposed [17,18,19,20,21,22,23,24,25,26,27,28,29,30,31,32,33,34,35,36,37,38]. Thus, CL methods can effectively reduce the training time of the network for new data adaptation, and they are used to reduce the cost of re-training or fine-tuning models in various fields in the real world. When a neural network is trained for the current task based on knowledge obtained from a previous task, it suffers from catastrophic forgetting and intransigence. Catastrophic forgetting implies the loss of important information from preceding tasks caused by incorporating new information relevant to the current task [17,22], and intransigence refers to the inhibition of learning information from the new task [18,22]. Thus, the goal of CL methods is to improve the performance of neural networks by alleviating catastrophic forgetting and avoiding intransigence.

Previous methods on overcoming catastrophic forgetting and intransigence can be classified into three categories: regularization-based methods, replay-based methods, and dynamic-architecture-based methods [17,18,19,20,22,39,40,41,42,43]. Regularization-based methods [23,24,25] alleviate catastrophic forgetting by preserving previous capabilities or imposing constraints when the weights of the neural network are updated. Replay-based methods [26,27,28,30,31,32,33,35,36,38,44] store examples from previous tasks in a memory buffer or reproduce old examples using a generative model, and, then, they train the network using both the old examples and the examples of the current task. Dynamic-architecture-based methods [34,45,46,47] improve performance by dynamically changing neural network resources by analyzing old examples and examples of the current task. Because the performance of CL methods changes according to the data of the current task and the data of the previous tasks, the scenario that defines the relationship between the current task and previous tasks is an important factor in CL.

Based on the assumption of the training data and given information, such as a task identifier (ID), various CL scenarios can be classified into three categories [17,18,20,22,41,42]: task incremental, class incremental, and domain incremental. The task-incremental scenario assumes that each task has a task ID, which indicates information on the class distribution and input distribution. The class-incremental scenario has the same setting as the task-incremental scenario; however, the task ID remains unknown. The domain-incremental scenario assumes that each task is set with different nonstationarity, different distributions of input data, and same class distribution. In general, class- and domain-incremental scenarios are more realistic and difficult because of the absence of a task ID.

From another viewpoint, CL scenarios are classified into offline and online approaches based on data accessibility of the current and previous tasks [22]. The offline approach of CL methods assumes that all data for the current task and previous tasks are accessible; however, the online approach of CL methods assumes that only one small batch is available, and that the previous batch data as well as the data of preceding tasks are not accessible. Considering that ASC tasks are suitable for mobile devices, which have limited computing power and memory resources, and because privacy of sound data is important for users, online approaches to CL methods are more practical.

Numerous studies have applied CL in various fields such as natural language processing (NLP) [19], human activity recognition (HAR) [39,40], and computer vision [22]; however, few studies have been conducted on ASC tasks. In addition, most CL studies on ASC tasks use task-incremental settings and offline approaches, which are relatively simple and unrealistic. In ASC tasks, class-incremental and domain-incremental scenarios are necessary to reflect real-world scenarios where sound classes are varied and device heterogeneity exists. In addition, for reasons discussed earlier, online approaches to CL methods are more suitable for ASC tasks. Therefore, in this study, we developed more realistic CL scenarios, such as online class-incremental (OCI) and online domain-incremental (ODI) scenarios, for various ASC datasets. Additionally, we systematically investigated the performance of recent CL methods using two proposed scenarios on three public datasets. Finally, we comprehensively analyzed the features of each CL method by comparing the performance of CL methods on various datasets.

This study makes the following contributions:

- We proposed OCI and ODI scenarios on three public datasets for evaluating the performance of CL methods in ASC tasks. For ODI scenarios, we used noise addition and SpecAugment to mimic the domain change. The public dataset contains the DCASE-2021 dataset [8], UrbanSound8K dataset [48], and ESC-50 dataset [49].
- Using the two scenarios on three public datasets, we systematically analyzed the performance of the recent CL methods. In the OCI scenario, we experimented with two regularization-based methods and eight replay-based methods. For the ODI scenario, we analyzed two regularization-based methods and seven replay-based methods, excluding iCaRL.
- We analyzed the experimental results using performance metrics such as accuracy and forgetting for both scenarios. Moreover, we evaluated the training time of the CL methods in accordance with the changes in buffer size for model training to validate the effectiveness of the CL methods.

The remainder of this paper is organized as follows. Section 2 reviews related works, and Section 3 describes the problem definition for online CL in ASC tasks. The CL methods are compared in Section 4. The experimental setup is presented in Section 5, and the experimental results are described in Section 6. Finally, conclusions and discussion are presented in Section 7.

## 2. Related Works

### 2.1. Acoustic Scene Classification

ASC involves the task of classifying a sound event into predefined classes such as acoustic scenes or acoustic environments [1,2,3,4]. Traditional methods for ASC tasks comprise feature extraction and classification models [2,3,4]. These methods first extract hand-crafted features such as the Mel-frequency cepstral coefficient (MFCC) and then classify the category of sound events using the extracted features and machine learning algorithms, such as support vector machines (SVMs) and K-nearest neighbors (KNN). However, these methods are limited in their ability to increase the classification performance.

To use the superior discriminative power of deep neural networks, deep-learning-based methods have been proposed for ASC tasks and have shown better classification performance than traditional methods. Most deep-learning-based methods use the convolutional neural network (CNN) architecture, and ResNet [9] is widely used as a backbone network in CNNs because of its high classification accuracy [10,11,12,13,50].

Although deep-learning-based methods show superior performance, they do not flexibly reflect the changes in sensor devices that gather sound events and the environments that determine the class of sound events. To deal with these changes that affect the performance of the trained network, re-training or fine-tuning of the networks, which incurs a high computational cost, is required.

CL involves a model that continually learns new tasks without forgetting the knowledge from preceding tasks [17,18,19,20,22,39,40,41,42,43]. CL can flexibly handle changes in tasks and incurs a lower computational cost than re-training or fine-tuning the network model. Although CL has gained considerable interest in various fields in recent years, few studies have focused [17,21] on ASC tasks to date.

### 2.2. Benchmark of Continual Learning Methods

Extensive research on CL is actively being conducted in the field of computer vision, and other fields such as NLP and HAR have received substantial attention. Thus, numerous methods have been proposed for CL; however, all these methods have reported different performances using different scenarios, different datasets for training the model, and different experimental environments. Therefore, there is a growing interest in benchmark studies to compare the performances of CL methods in a fair manner.

In a previous study by [17], biological research and a complete learning system were compared based on the plasticity and stability of the human brain. In addition, the performances of the CL methods, including traditional methods and deep-learning-based methods, were summarized for image and audio classification tasks. Another study benchmarked CL methods for image classification tasks in the field of computer vision [22]. In addition, refs. [39,40] applied regularization-based and replay-based methods to HAR tasks and proposed a framework to evaluate the performance of such techniques on various public HAR datasets. Ref. [19] compared the performance of CL methods for various NLP tasks, including word and sentence representations, language modeling, and question answering. Although studies comparing the performance of CL methods have been conducted in various fields, a comprehensive benchmark study on ASC tasks is yet to be conducted.

CL scenarios constitute one of the most important factors in benchmark studies on CL methods because they determine the accessibility to the current and previous tasks and prior knowledge, such as the task ID. Widely used scenarios for CL are task-, domain-, and class-incremental scenarios [41].

In addition, a previous study [20] used an offline approach that can access all data for the current and previous tasks multiple times. However, these settings are unrealistic; therefore, a recent benchmark study [22] was conducted using more realistic scenarios. It used an online approach that can access only one small batch and cannot access the previous batch data and data of the preceding tasks. Another ASC study [21] using a task-incremental setting and an offline approach has also been reported. However, considering the features of sound data, such as privacy issues and device heterogeneity, a benchmark study using incremental class and domain settings and an online approach is necessary. Further, it is necessary to explore the performance of CL methods in ASC tasks because the characteristics of sound data are different from those of language, sensor, and image data. Thus, in this paper, we propose more realistic and difficult scenarios, such as OCI and ODI, for various publicly available sound datasets.

## 3. Problem Definition for Online Continual Learning in Acoustic Scene Classification Tasks

The performance of CL methods is significantly influenced by the scenarios that define the problem. Therefore, in this section, we describe the scenarios for the ASC tasks.

**Figure 1:**
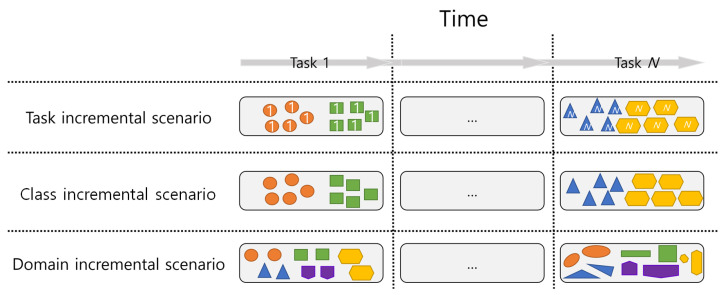
Conceptual illustration of the three types of CL scenarios. The task-incremental scenarios only have a task ID per data. The class-incremental scenarios have different input data and class distributions without the task ID. The domain-incremental scenario assumes that each task is set with different nonstationarity, different distributions of input data, and the same class distribution.

CL scenarios have a sequential set of tasks, $T=\left\{T_{1},\cdots ,T_{t}\right\}$. At time *t*, CL methods receive the data and information on task *t*. According to the input data and information, typical CL scenarios can be categorized into three types: task-, class-, and domain-incremental scenarios. A conceptual illustration of the three CL scenarios is depicted in Figure 1. As shown in Figure 1, task-incremental scenarios have a task ID as prior information, unlike in the other scenarios. At time *t* in task-incremental scenarios, data distribution, $D_{t}=\left\{\left(x_{1},y_{1}\right),\cdots ,\left(x_{n},y_{n}\right),task\_ID\right\}$, consists of the task ID and *n* samples with input data *x* and label *y*. Because the neural network cannot recognize the task ID in the real world, the task-incremental scenario is unrealistic. In the case of the class-incremental scenario, each task takes different input data and class distribution without a task ID. Data distributions of the class-incremental scenario are the same as those of task-incremental scenarios without the task ID, $D_{t}=\left\{\left(x_{1},y_{1}\right),\cdots ,\left(x_{n},y_{n}\right)\right\}$. Without the task ID, class-incremental scenarios are more difficult and realistic compared with the task-incremental scenarios. In the domain-incremental scenario, each task takes different input data but may have the same class distribution. Data distributions of domain-incremental scenarios are represented by $D_{t}=\left\{\left(x_{1}^{{}^{\prime }},y_{1}\right),\cdots ,\left(x_{n}^{{}^{\prime }},y_{n}\right)\right\}$, where $x^{{}^{\prime }}$ represents a different input augmented from a previous task and *y* represents the same label with a previous task. To mimic a domain change that has different input data for the same class, data-augmentation techniques such as noise addition and SpecAugment [51,52] are used for domain-incremental scenarios.

**Figure 2:**
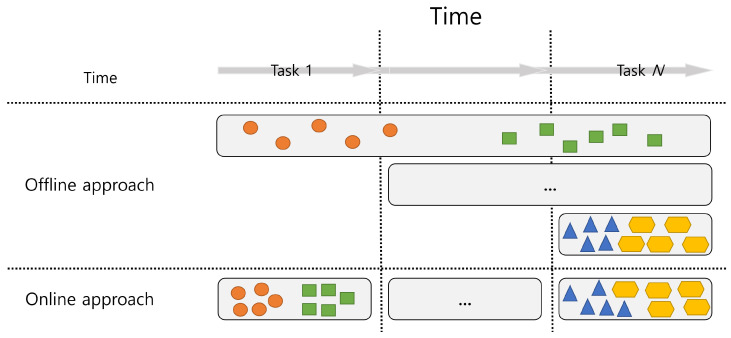
Conceptual illustration of the offline and online approaches for class-incremental scenarios. The offline approach uses all data from previous tasks while training a neural network with a new task. In contrast, the online approach uses only new tasks.

CL scenarios are also classified into offline and online approaches based on data accessibility for current and previous tasks during model training. A conceptual illustration of the offline and online approaches for class-incremental scenarios is shown in Figure 2. As shown in Figure 2, the offline approach for CL can access all data from the first task to the current task, *T*, when training the network model at the time of task *T*. However, the online approach for CL can only use the current batch data of task *T* when training the network model for task *T*.

Recently, applications of ASC tasks have received substantial attention for real-time sound recognition [5,7] and sound-based monitoring systems for smart factories [6]. Realistic CL scenarios that require limited computing resources and no prior information are necessary for such applications. Therefore, in this paper, we propose OCI and ODI scenarios for various public datasets to consider real scenarios.

## 4. Comparison of Continual Learning Methods

Existing CL methods can be classified into three categories: regularization-based, replay-based, and dynamic architecture-based. Only a few studies using dynamic architecture-based methods have been conducted for online scenarios. In addition, dynamic architecture-based methods require considerable time to adapt the network structure to the current task dynamically. Therefore, in this study, we focused on regularization-based and replay-based methods.

The network architecture can vary depending on the CL scenario [25]. For task-incremental scenarios, a neural network with a multihead architecture has been used [36,39,40]. In contrast, a neural network with a single-head architecture has been used to evaluate the performance of CL methods for class- and domain-incremental scenarios [22,38,44]. Therefore, we used a single-head architecture for all CL methods and modified the network to apply online scenarios.

### 4.1. Regularization-Based Methods

Regularization-based methods alleviate catastrophic forgetting by preserving weights important for previous tasks while updating the weights of the neural network. Representative methods include elastic weight consolidation (EWC) [24] and learning without forgetting (LwF) [23].

EWC mitigates catastrophic forgetting by selectively decelerating the update of weights according to the impact of previous works. The Fisher information matrix is used to define the importance of the network parameters, and the regularization loss with the Fisher information matrix is used to update the parameters for the new task while maintaining the parameters learned in the previous tasks. Given network parameters θ, *A*, and *B*, the loss function of EWC is defined as [24]

(1) $$L\left(\theta \right)=L_{B}\left(\theta \right)+\sum_{i}^{}\frac{\lambda }{2}F_{i}{\left(\theta_{i}-\theta_{A,i}^{*}\right)}^{2}\;$$

where *F*, *i*, λ, and $L_{B}\left(\theta \right)$ represent the Fisher information matrix, each parameter, weight when considering the previous task, and loss for task *B*, respectively. However, EWC cannot be applied directly to online scenarios [22], and, therefore, we used EWC++ [25], which can set online scenarios for the experiments.

For efficient computation and memory usage, EWC++ maintains a single diagonal Fisher matrix, *F*, and uses the moving average to update the Fisher matrix.

(2) $$F_{\theta }^{t}=\alpha F_{\theta }^{t}+\left(1-\alpha \right)F_{\theta }^{t-1}$$

where EWC++ calculates $F_{\theta }^{t}$ with the current batch and α is a hyperparameter between 0 and 1. The Fisher matrix computed by EWC++ eliminates an additional pass over the dataset, and thus it can efficiently use memory by only storing two sets of the Fisher matrix.

LwF [23] trains a neural network with only new task data and uses knowledge distillation to preserve previous capabilities. The output of the old network is computed using new task data, which are used as soft labels for knowledge distillation. Given that the network parameter, θ, consists of shared parameters, task-specific parameters for each task, and parameters for the current tasks, the loss function of LwF is defined as [23]

(3) $$L\left(\theta \right)=\lambda L_{old}\left(Y_{o},{\hat{Y}}_{o}\right)+L_{new}\left(Y_{n},{\hat{Y}}_{n}\right)+R\left(\theta \right)\;$$

where $Y_{o}$, $Y_{n}$, ${\hat{Y}}_{o}$, and ${\hat{Y}}_{n}$ represent the output of the old network for the new task data, ground truth for the new task data, predictions of the current network for old tasks, and predictions of the current network for current tasks, respectively. λ represents the weight that sets the degree to which a previous task affects a new task.

### 4.2. Replay-Based Methods

Replay-based methods store the information of the examples of preceding tasks in a memory buffer [29,30,31,32,33,35,36,38,44] or generative model [26,27]. Then, examples of the preceding tasks and new data for the current task are used together to train the network model for the current task. Therefore, replay-based methods consist of three steps: memory retrieval, model update, and memory update. In the memory-retrieval step, old examples from the stored information are retrieved using memory-retrieval algorithms. Then, in the model-update step, the parameters of the network model are trained using old examples and new data for the current task. Finally, in the memory-update step, some new task data that have representative features for the current task are stored in the memory buffer or reflect the generative model by training the network model.

Training the network model using all older examples and new data can mitigate catastrophic forgetting; however, storing all examples is inefficient because of the limited memory space and data redundancy. Thus, experience replay (ER) [28,35] uses a sampling method to select a limited number of examples. The reservoir sampling method was used to replace the old samples in the memory-update step, and the random sampling method was used to select the old samples in the memory buffer. In addition, ER uses cross-entropy loss to train the neural network with new data and old examples stored in the memory buffer. Recently, Ref. [35] highlighted that, although ER is simple, it exhibits superior performance compared with conventional CL methods.

Greedy sampler and dumb learner (GDumb) [31] was originally designed to solve the open-set recognition problem; however, it also solves the CL problem with superior performance. GDumb comprises a greedy sampler and a dumb learner. When new task data arrive, the greedy sampler manages the memory buffer to maintain the balance of the sample by category. The dumb learner trains the neural network from scratch using examples provided by the greedy sampler.

Gradient episodic memory (GEM) [32] uses the information of the loss gradient vector calculated using examples in the memory buffer to decide whether to update the parameters. Because GEM calculates the loss gradient vector for each previous task, it incurs considerable computational cost. In contrast, averaged gradient episodic memory (A-GEM) [33] uses the average loss gradient vector calculated for all previous tasks to reduce the computational costs. From the viewpoint of network training, GEM tries to decrease the loss of examples per task, whereas A-GEM tries to decrease the average loss of examples for all tasks. We used A-GEM, which is an efficient version of GEM, to compare the performance of various CL methods.

Gradient-based sample selection (GSS) [29] also formulates the CL problem as a constrained optimization problem, similar to GEM and A-GEM. However, GEM and A-GEM focus on the model-update step, whereas GSS focuses on the memory-update step by retrieving examples with a sample selection algorithm formulated as a constraint reduction problem. GSS selects examples that maximize the diversity of samples in the memory buffer by analyzing the parameter gradient. Two online sample selection algorithms were proposed for the GSS: GSS-integer quadratic programming (IQP) and GSS-greedy. In this study, we adopted the GSS-greedy method used in [22] for our experiments.

Incremental classifier and representation learning (iCaRL) [36] introduces the nearest-class-mean (NCM) classifier [53] for class-incremental learning and has a superior classification accuracy because it uses the mean of exemplar images robust to small changes in data. iCaRL manages memories for replay so that exemplars have priority, and the number of exemplars in each class is kept the same. To train the network model, the loss, consisting of classification and distillation terms [37], is calculated using exemplar sets and new task data. The labels of the new task data are classified by comparing the distance between the average feature vectors of all exemplars for each class and the input feature vector.

Maximally interfered retrieval (MIR) [30] was proposed to increase the performance of the CL method by improving the memory sampling method for replay. Unlike random sampling, which is a widely used sampling method for memory retrieval, MIR reduces catastrophic forgetting by selecting samples that interfere the most with new tasks from among the samples of old tasks used for training the network model. MIR can be applied to both the experience replay method and generative replay method, and can be used with other replay-based methods such as ER, GEM, and iCaRL. In this study, we used ER with MIR as the MIR method for the experiments.

Adversarial Shapley value experience replay (ASER) [38] introduced a method that can evaluate the effect of samples in terms of the performance of the training model and used them in the memory-retrieval step. ASER uses the Shapley value (SV) to measure the contribution of each data point; the SV is calculated by measuring the marginal improvement in the classification accuracy as each sample is added. Because the SV requires computational complexity, $O(N^{2}$), to calculate the training dataset, ASER uses the KNN-SV method [54] that can be computed in O(NlogN) to speed up the computation of SV. ASER uses the adversarial SV (ASV), containing the cooperative and adversarial terms, as [38]

(4) $$ASV\left(i\right)=\max_{j\in S_{sub}}s_{j}\left(i\right)-\min_{k\in B_{n}}s_{k}\left(i\right)$$

where $S_{sub}$ represents the subset of memories, $B_{n}$ represents the current batch data, and $s_{j}\left(i\right)$ denotes the marginal contribution of each sample, *i*, with regard to *j*. In Equation (4), the first term is related to the cooperative notion that has a high positive SV for data in the memory buffer; the second term is related to the adversarial notion that has a negative SV with a large magnitude for new task data.

Replay-based methods suffer from class imbalance between the preceding and current tasks. SoftMax classifiers cannot effectively deal with the problem of class imbalance. Thus, supervised contrastive replay (SCR) [44] substitutes SoftMax classifiers with an NCM classifier [53] and introduces supervised contrastive loss to create better clusters in the feature space of the NCM classifier. The experimental results of SCR show that it outperforms the existing methods in online CL.

## 5. Experiments

### 5.1. Acoustic Scene Classification Datasets

To evaluate the performance of the CL method on ASC tasks, we used three public datasets of ASC tasks: DCASE-2021 [8], UrbanSound8K [48], and ESC-50 [49]. Table 1 lists the detailed information of the three datasets.

DCASE-2021 is an audio dataset used for classifying acoustic scenes in Task 1 A of the DCASE 2021 challenge. It has 10 classes, and the numbers of samples for training and validation are 13,962 and 2970, respectively. We pre-processed the audio data in a manner similar to that in [55]. We calculated 128 log-Mel energies under the original sampling rate of 44.1 kHz for each time slice by taking 2048 FFT points with a 50% overlap. After pre-processing, the input size of the audio data was 423 × 128 × 3. In DCASE-2021, we compared the methods using various memory buffer sizes including 200 (0.2k), 500 (0.5k), 1000 (1k), 2000 (2k), and 5000 (5k). In addition, we used the OCI/ODI scenarios to evaluate the performance of all the aforementioned CL methods on the DCASE dataset.

UrbanSound8K is a widely used dataset for evaluating the performance of the environmental sound classification methods, and contains 8732 labeled environmental sound clips of up to 4 s for 10 classes. To pre-process audio data, we ensured that all audio clips were 4 s long through zero padding, as in [56,57]. We extracted features with a 128-dimensional log-Mel filterbank at a sampling rate of 11,025 Hz for each time slice by taking 2048 FFT points with a 50% overlap. For UrbanSound8K, we used the same settings as for DCASE-2021 for the size of the memory buffer and online scenarios.

ESC-50 is a dataset used for classifying environmental sounds, and consists of 2000 labeled environmental recordings equally balanced between 50 classes of 40 clips per class. We divided the ESC-50 into 1400 audio clips for training and 600 audio clips for evaluation. Because the total number of samples for ESC-50 was 2000, a memory buffer larger than 2k was not adequate for an online scenario setting. Therefore, we experimented with the compared methods to obtain memory buffer sizes of 0.2, 0.5, and 1k. We pre-processed the dataset similar to [56,57], sampled the audio at 32k Hz, and converted the audio waveform to a sequence of 128-dimensional log-Mel filterbank features computed with a 25 ms Hamming window every 10 ms. We used the ODI scenario only for ESC-50.

### 5.2. Scenarios of Continual Learning for Acoustic Scene Classification Tasks

Considering the features of sound data, such as privacy issues, the online approach is appropriate for ASC tasks because it accesses only one small batch and cannot access the previous batch data and data of preceding tasks. In addition, class- and domain-incremental scenarios are useful for considering the characteristics of sound data, which can vary depending on the microphone of the devices and data-gathering environments. Thus, in this study, we used more realistic and difficult scenarios, such as OCI and ODI, to evaluate the performance of the CL methods.

#### 5.2.1. OCI Scenario

DCASE-2021 and UrbanSound8K with 10 classes were divided into five tasks, with two classes for each task. ESC-50 with 50 classes was split into 10 tasks, and each task contained five classes.

#### 5.2.2. ODI Scenario

The datasets for domain-incremental scenarios used in computer vision were permutated MNIST and Cifar datasets with a nonstationary distribution caused by noise, blur, and occlusion [22]. To emulate the domain change in the sound data, we applied data-augmentation techniques such as noise addition and SpecAugment [51,52] for the ODI scenarios. For each ODI scenario, we divided the five tasks into DCASE-2021 and UrbanSound8K.

### 5.3. Evaluation Metrics

To evaluate the performance of CL methods in the two CL scenarios, we measured the accuracy and extent of forgetting per task and calculated the average accuracy and forgetting for all tasks, given that the accuracy of task *i*, $a_{T,i}$ is trained on task *T*, and the average accuracy, $A_{T}$, is defined as [22]$A_{T}=\frac{1}{T}\sum_{i=1}^{T}a_{T,i}$.

Average forgetting measures the degree of forgetfulness of each preceding task after training the neural network for the last task, *T*. The average forgetting, $F_{T}$, was calculated using [22]$F_{T}=\frac{1}{T-1}\sum_{i=1}^{T-1}f_{T,i}$, where $f_{T,i}=\max_{k\in \left\{1,\cdots ,t-1\right\}}a_{k,i}-a_{T,i}$.

Because the average accuracy is related to the intransigence and the average forgetting is related to catastrophic forgetting, we can evaluate the performance of the CL methods using the two metrics.

### 5.4. Implementation Details

ResNet is widely used as a backbone network for classifying acoustic scenes and has demonstrated superior performance [10,11,12,13]. Hence, we adopted the reduced ResNet-18 model, considering the lack of memory and long training time [22,38,44]. We set the batch size to 10 and used the stochastic gradient descent optimizer with a tuned learning rate to train the reduced ResNet-18 model. We use single-head architecture with output units for all classes to experiment with the CL method in the OCI and ODI scenarios. As the first task, we tuned the learning rate with various learning rates set from 0.0001 to 0.1. Then, we trained the reduced ResNet-18 model with a tuned learning rate from the first task.

To compare the performance of CL methods on ASC tasks, we modified the benchmark framework of CL for computer vision tasks [22,44]. To tune the hyperparameters without violating the online CL setting, previous studies [22,33] separated the data streams for cross-validation and for training and evaluation. They used the data stream of the first and second tasks for tuning the hyperparameters, and the data stream of the other tasks for evaluating the performance. Similar to previous studies, we used the data stream of the first and second tasks for tuning the hyperparameters. However, we evaluated the performance from the first task to the last task using optimal hyperparameters because this is more suitable for an online setting and yields results similar to realistic scenarios.

## 6. Experimental Results

We evaluated the performance of the three baseline methods to compare the CL methods with conventional methods: offline, online, and fine-tuning. The offline method trains the network model using all data of the dataset for 50 epochs with a mini-batch size of 128, and this provides the upper bound of the performance for the CL methods. The online method trains the network model using all data of the dataset for one epoch with a mini-batch size of 10; all data of the dataset can be seen once. The fine-tuning method always updates the network model for the current task and provides a lower-bound performance for the CL methods. For the OCI scenario, we analyzed ten recent CL methods comprising two regularization-based methods and eight replay-based methods. In the ODI scenario, we analyzed nine CL methods excluding iCaRL, because iCaRL is not suitable for the ODI scenarios. iCaRL updates the model with distillation loss calculated from the data of old classes; however, the ODI scenarios have different input data for the same class from all tasks.

### 6.1. Performance Comparison in the Online Class-Incremental Scenario

#### 6.1.1. Overall Analysis

From our studies, we showed that the regularization-based methods demonstrated a performance in terms of accuracy similar to that of the fine-tuning methods, providing a lower-bound performance; however, LwF showed the best performance in terms of forgetting compared with other methods, including the replay-based methods. This result indicates that LwF is good for catastrophic forgetting, but not for intransigence.

The replay-based methods showed superior performance compared with the baseline and regularization-based methods. GDumb, iCaRL, and SCR showed superior performance in terms of accuracy compared with other methods. This tendency is similar to the benchmark results of the CL methods for computer vision tasks [22]. These experimental results show that we must consider both accuracy and forgetting metrics to devise a CL method for ASC tasks.

#### 6.1.2. Average Accuracy

Table 2 summarizes the experimental results of the average accuracy for the OCI scenario in ASC tasks. For the three datasets, the replay-based methods showed higher accuracies compared with the regularization-based methods, and the regularization-based methods had an accuracy similar to that of the fine-tuning methods, which provide lower-bound performance. Among the replay-based methods, iCaRL, GDumb, and SCR had higher accuracies compared with the other replay-based methods. If the size of the memory buffer was large in the three datasets, GDumb had the highest accuracy. The experimental result of GDumb for a buffer size of over 0.5k in the ESC-50 dataset shows that GDumb had the best performance by a large margin. For small buffer sizes (0.2k and 0.5k), iCaRL had the best accuracy in DCASE-2021, and SCR had the best accuracy in UrbanSound8K. In addition, in ESC-50, the performance of SCR for a 0.2k buffer size was the best. Among the replay-based methods, A-GEM showed the lowest performance for all sizes of memory buffers in all datasets and it was similar to the baseline methods and regularization-based methods. This tendency is similar to the benchmark results of the CL methods for computer vision tasks [22]. Although MIR has the best performance in terms of accuracy in CORe-50-NC for image classification tasks [22], it did not show impressive results in the ASC tasks. Experimental results indicate that the performance of the numerous methods was not affected by the size of the memory buffer; however, the performance of GDumb increased in proportion to the size of the memory buffer. Of all CL methods, GDumb had the best performance, which was similar to that of offline methods with the largest buffer size in all datasets. In addition, the size of the memory buffer with the highest accuracy differs depending on the method. Therefore, the size of the memory buffer is another important parameter to consider when deploying CL methods for the ASC tasks.

#### 6.1.3. Average Forgetting

Table 3 presents the experimental results of the average forgetting for the OCI scenario in the ASC tasks. Average forgetting is related to average accuracy because average forgetting indicates the extent to which the network model forgets each preceding task after being trained on the last task, *T*. A previous study [22] on computer vision tasks showed that a method with high accuracy shows lower forgetting, and this is similar to our experimental results. Among the replay-based methods for ASC tasks, SCR, iCaRL, and GDumb showed superior performance in terms of average accuracy and forgetting. In most cases, SCR exhibited the best performance in terms of average forgetting among the replay-based methods. iCaRL had the highest accuracy and lowest forgetting in the case of a buffer size of 0.2k on DCASE-2021. GDumb had the highest accuracy and lowest forgetting when the memory buffer sizes were 2k and 5k for UrbanSound8K. In DCASE-2021 and ESC-50, LwF had the lowest average forgetting among the compared methods; however, it had lower performance in terms of average accuracy. This result indicates that LwF is good for catastrophic forgetting, not intransigence. EWC showed a performance in terms of average forgetting similar to that of the fine-tuning method. The experimental results of the average accuracy showed that ASER, ER, GSS, A-GEM, and MIR did not show good performance in terms of average forgetting. The experimental results showed that LwF could alleviate only intransigence, and GDumb, iCaRL, and SCR could significantly reduce both catastrophic forgetting and intransigence. Therefore, we must consider both the accuracy and forgetting metrics to devise a CL method for ASC tasks.

### 6.2. Performance Comparison in Online Domain-Incremental Scenario

#### 6.2.1. Overall Analysis

In the two ODI scenarios, the performance gap between the CL methods and fine-tuning methods was lower than that of the OCI scenario, which indicates that the ODI scenario is more difficult than the OCI scenario. In ODI scenarios, regularization-based methods have similar performances in terms of the average accuracy of the fine-tuning method. However, both LwF and EWC perform better than the fine-tuning method. This implies that regularization-based methods such as LwF and EWC are suitable for catastrophic forgetting in the ODI scenario. Most replay-based methods, except for A-GEM, have performances in terms of average accuracy and average forgetting that are superior compared with the fine-tuning method. As in the experimental results for the OCI scenario, GDumb had the highest accuracy when used with a large-size memory buffer in DCASE-2021; however, A-GEM had a lower accuracy compared with the regularization-based methods and fine-tuning method in DCASE-2021 and UrbanSound8K. SCR consistently outperformed the compared methods in terms of accuracy. These experimental results show that we can consider SCR first in ODI scenarios.

#### 6.2.2. Average Accuracy

Table 4 and Table 5 list the experimental results of the average accuracy for ODI scenarios in DCASE-2021 and UrbanSound8K, respectively. In DCASE-2021 and UrbanSound8K, the average accuracy of regularization-based methods, including LwF and EWC, was similar to that of the fine-tuning method. This result showed a tendency similar to that of previous work [22] for image classification tasks. However, replay-based methods have different tendencies. ER and MIR have shown impressive performance, which outperforms the fine-tuning method in image classification tasks; however, they do not show the best performance in ASC tasks. In contrast, GDumb and SCR have shown superior performance compared with other methods, unlike the results of image classification tasks. In DCASE-2021 with a 5k buffer size, GDumb had the best performance among all CL methods. The SCR consistently outperformed the compared methods in terms of accuracy in most cases, except for DCASE-2021 with a 5k buffer size. SCR with a 5k memory buffer had higher accuracy compared with the offline method on UrbanSound8K. SCR adopts supervised contrastive learning, and, therefore, augmenting the training data is effective for ODI scenarios with a different distribution of input data for the same class. In addition, GDumb had the highest accuracy with a 5k memory buffer in the two types of scenarios on the DCASE-2021. In most cases, the A-GEM had a lower average accuracy compared with the fine-tuning method. Similar to the results of the OCI scenario, the A-GEM had a lower performance compared with the fine-tuning method. ASER and GSS did not perform well in the ODI scenarios. From the experimental results on average accuracy, we must first consider SCR and GDumb.

#### 6.2.3. Average Forgetting

Table 6 and Table 7 list the experimental results of the average forgetting for ODI scenarios in DCASE-2021 and UrbanSound8K, respectively. As shown in Table 3, LwF exhibited lower average forgetting compared with the fine-tuning method. Although the average forgetting of EWC in the OCI scenario showed lower performance, EWC in the ODI scenario showed good performance, which was similar to that of LwF. Regularization-based methods such as LwF and EWC had relatively lower performance, similar to the results [44] of image classification tasks. Thus, the regularization-based method in the ODI scenario is suitable for catastrophic forgetting. Replay-based methods exhibit various performances, from bad to good. Our results indicate that A-GEM, ASER, and ER showed lower performances compared with replay-based methods, and this tendency is similar to the results of the OCI scenario. For small sizes (0.2k), MIR exhibited good performance in the noise of DCASE-2021. This result is similar to that of the OCI scenario. Unlike the results of the OCI scenario among the replay-based methods, GSS for 0.2k, 0.5k, and 1k in SpecAugment of DCASE-2021 showed a higher performance in terms of average forgetting. The average forgetting of GSS for 0.2k shows that its performance is the best among all CL methods in DCASE-2021. The SCR outperformed the compared methods in terms of the average forgetting in most cases of ODI scenarios, whereas LwF outperformed the compared methods in terms of the average forgetting in the OCI scenario. Considering both the average accuracy and average forgetting, we found that SCR is the most suitable method for ODI scenarios.

### 6.3. Training Time

We measured the training time of each CL method because training time is a key factor for assessing the performance of CL methods. For a fair comparison, we excluded the time for grid search because each method has a different search space, which significantly affects the search time. We experimented with CL methods with all memory buffers from 0.2k to 5k and applied the OCI scenario on the DCASE-2021 dataset. The average training time was calculated by taking the average over the sum of the training times; this was repeated three times on an NVIDIA RTX 8000 GPU.

**Figure 3:**
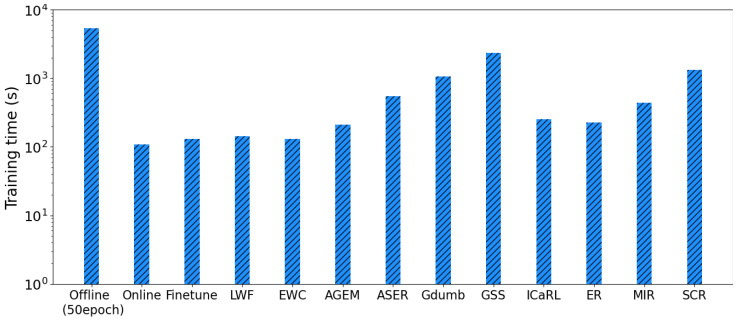
Comparison of training times for different models in the case of a memory buffer size with 1k. The training time is plotted on a logarithmic scale. Offline is the upper bound of the training time. Baseline and regularization-based methods have lower training times compared with replay-based methods. With a 1k memory buffer size, GSS, GDumb, and SCR have higher training times.

The average training time, set to a memory buffer size of 1k, for CL methods on DCASE-2021 is shown in Figure 3. In Figure 3, the training time is plotted on a logarithmic scale, and the offline method using 50 epochs had the longest training time compared with the other methods. Replay-based methods have a longer training time compared with regularization-based methods because replay-based methods need to manage the memory buffer to retrieve old samples or update the samples. Methods with superior performances such as GDumb and SCR have a longer training time among the replay-based methods. iCaRL showed good performance in terms of both accuracy and training time, whereas GSS showed lower performance in both accuracy and training time. The experimental results showed that the average accuracy of the CL methods was highly related to training time, except in the case of GSS.

A previous study [22] showed only the training time for the compared methods on a specific memory buffer size. However, the size of the memory buffer is a very important factor for determining the performance of replay-based methods, as shown in Section 6.1 and Section 6.2. Therefore, we assessed the training time of the replay-based methods for various sizes of memory buffers. We applied the OCI scenario to DCASE-2021 to measure the training time.

**Figure 4:**
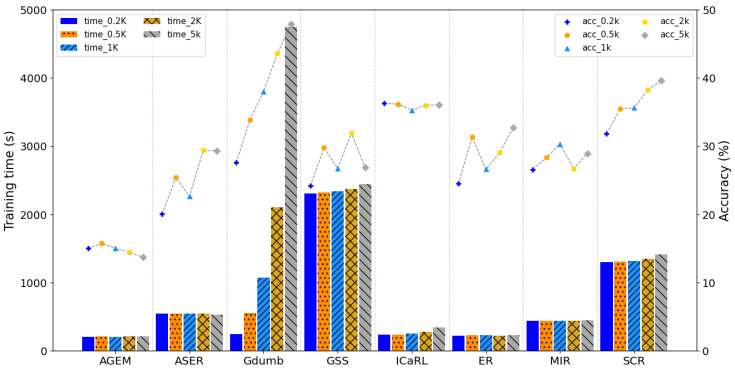
Averaged training time and accuracy of different memory-based methods according to changes in the size of the memory buffer. The vertical bar indicates the averaged training time, and dots represent the accuracy of each model according to the buffer size. For easy identification, we used different colors according to the size of the memory buffer.

The average training time and accuracy of the replay-based methods with all memory buffer sizes from 0.2k to 5k are shown in Figure 4. In Figure 4, the experimental results show that ASER, ER, and MIR had a constant training time, regardless of the size of the memory buffer; their performance increased with an increase in the size of the memory buffer. The training time of ASER was slightly reduced in proportion to the size of the memory because the large buffer size reduced the number of memory updates.

In contrast, A-GEM had a constant training time for the size of the memory buffer; however, the performance of A-GEM decreased with an increase in the size of the memory buffer. The training times of GSS, iCaRL, and SCR slightly increased with an increase in the size of the memory buffer; however, their performance irregularly increased or decreased by a large margin with an increase in the size of the memory buffer.

Although the average accuracy of GDumb increased in proportion to the size of the memory buffer, the training time of GDumb increased significantly according to the memory buffer size. The training time of GDumb was 242 s when the memory buffer size was 0.2k; however, the training time of GDumb increased to 4753 s when the memory buffer size increased to 5k.

## 7. Conclusions

We used CL for ASC tasks for realistic scenarios considering the features of sound data such as privacy issues and device heterogeneity. The online approach that accesses only one small batch and cannot access the previous batch data and data of preceding tasks is appropriate for ASC tasks because of privacy issues; hence, the proposed scenarios are based on an online approach. The proposed scenarios contain OCI and ODI scenarios that reflect the device heterogeneity and change in the data-gathering environments. We defined OCI and ODI scenarios using three public datasets: DCASE-2021, UrbanSound8K, and ESC-50. With the two proposed scenarios on three public datasets, we systematically analyzed the performance of ten recent CL methods (two regularization-based methods and eight replay-based methods) for the OCI scenario, and nine CL methods (except for iCaRL). iCaRL, which requires both old and new classes to use knowledge distillation, is not suitable for ODI scenarios that have the same class for all tasks.

In the OCI scenarios, regularization-based methods showed a performance similar in terms of accuracy to the fine-tuning method, which provides a lower-bound performance; however, LwF showed the best performance in terms of forgetting compared with the other methods, which includes replay-based methods. Replay-based methods showed superior performance compared with the baseline and regularization-based methods. GDumb, iCaRL, and SCR showed superior performance in terms of accuracy compared with the other methods; this tendency is similar to the benchmark results of the CL methods for computer vision tasks. In the ODI scenarios, regularization-based methods showed a performance similar in terms of average accuracy and average forgetting to that of the fine-tuning method. Most replay-based methods had superior performance in terms of the average accuracy and average forgetting compared with the fine-tuning method. In the two ODI scenarios on the two datasets, SCR consistently outperformed the compared methods in terms of accuracy. In addition, we analyzed the training time of each replay-based method for various memory buffer sizes. Most replay-based methods containing ASER, ER, and MIR had almost constant training times, regardless of the size of the memory buffer; their performance increased with an increase in the size of the memory buffer. In addition, the training times of GSS, iCaRL, and SCR slightly increased with an increase in the memory buffer size. Although the average accuracy of GDumb increased in proportion to the size of the memory buffer, the training time of GDumb significantly increased with an increase in the memory buffer size.

We focused on analyzing the performance of regularization-based and replay-based methods for ASC tasks. Because dynamic-architecture-based methods are representative methods for CL, we plan to analyze the performance of dynamic-architecture-based methods for ASC tasks. The experimental results of this study indicate that most replay-based methods with superior accuracy suffer from a long training time. Therefore, in future work, we plan to devise an efficient replay-based method that has a short training time for various memory buffer sizes and superior performance in terms of accuracy and forgetting.

## Figures and Tables

**Table 1:** Detailed information of the ASC datasets. The attributes include the number of classes, tasks, and total data. The number of classes per task is 2 in DCASE-2021 and UrbanSound8K, and 5 in ESC-50. The information contains the input size and online scenarios for experiments. DCASE-2021 and UrbanSound8K experiments with CL methods are under OCI and ODI scenarios, but ESC-50 experiments with CL methods are under only the OCI scenario.

Dataset	DCASE-2021	UrbanSound8K	ESC-50
#Classes	10	10	50
#Task	5	5	10
#Total data	23,040	8732	2000
Input size	423 × 128 × 3	44 × 60 × 1	512 × 128 × 1
Online scenarios	OCI/ODI	OCI/ODI	OCI

**Table 2:** Average accuracy (%) of the CL methods in the OCI scenario. A higher score is better.

Dataset	DCASE-2021					UrbanSound8K					ESC-50		
Offline	55.33					89.78					59.96		
Online	38.46					60.57					10.53		
Fine-tuning	16.39					17.28					3.17		
LwF	15.45					17.67					4.53		
EWC	16.10					15.91					4.61		
Buffer Size	0.2k	0.5k	1k	2k	5k	0.2k	0.5k	1k	2k	5k	0.2k	0.5k	1k
A-GEM	15.01	15.76	15.01	14.50	13.72	18.02	18.15	17.25	17.35	16.91	5.27	5.61	3.99
ASER	20.07	25.40	22.65	29.37	29.34	40.27	48.53	51.72	58.27	56.08	10.14	10.90	11.35
GDumb	27.59	33.86	**38.03**	**43.58**	**47.94**	47.38	61.09	**69.26**	**78.61**	**87.42**	25.61	**41.16**	**54.42**
GSS	24.18	29.78	26.77	31.94	26.88	38.93	46.50	52.00	49.52	53.11	11.29	9.78	12.02
iCaRL	**36.28**	**36.18**	35.28	36.02	36.05	50.77	52.21	55.56	52.33	52.73	16.13	21.80	23.29
ER	24.50	31.34	26.61	29.08	32.74	39.91	48.00	53.31	45.31	48.63	10.71	8.95	11.48
MIR	26.59	28.31	30.33	26.70	28.96	33.62	50.98	49.66	53.66	54.62	10.57	11.83	10.22
SCR	31.85	35.48	35.63	38.24	39.61	**56.73**	**61.42**	62.49	64.57	65.62	**25.90**	34.72	37.70

**Table 3:** Average forgetting (%) of CL methods in the OCI scenario. A lower score is better.

Dataset	DCASE-2021					UrbanSound8K					ESC-50		
Fine-tuning	60.14					71.62					27.95		
LwF	**4.35**					**7.41**					**2.01**		
EWC	62.44					65.54					35.48		
Buffer Size	0.2k	0.5k	1k	2k	5k	0.2k	0.5k	1k	2k	5k	0.2k	0.5k	1k
A-GEM	60.19	62.86	60.19	63.77	64.32	69.07	70.69	63.35	70.85	67.50	40.03	40.23	39.73
ASER	52.82	53.50	49.97	44.22	43.12	46.63	39.06	32.32	23.86	30.04	33.33	33.32	38.06
GDumb	22.58	23.28	20.87	18.54	19.48	19.46	15.62	13.31	**7.93**	**4.02**	24.27	19.23	12.32
GSS	48.21	46.36	43.94	31.32	40.87	48.30	37.49	25.99	32.79	31.15	32.06	33.49	31.74
iCaRL	**16.88**	18.41	16.00	**13.61**	13.26	19.35	14.96	14.36	14.30	16.09	14.49	10.79	10.05
ER	49.50	39.30	39.42	44.21	35.16	44.99	35.99	31.26	37.00	37.00	36.28	35.49	32.70
MIR	40.56	35.00	29.59	33.88	33.86	51.57	27.83	27.54	22.90	20.61	32.84	31.56	32.07
SCR	17.61	**12.02**	**13.93**	13.67	**10.84**	**15.63**	**12.11**	**11.49**	9.08	7.62	**14.28**	**9.14**	**9.08**

**Table 4:** Average accuracy (%) of the CL methods in DCASE-2021 in the ODI scenario. A higher score is better.

Dataset	DCASE-2021									
ODI scenario	Noise					SpecAugment				
Offline	46.35					50.43				
Online	27.08					28.36				
Fine-tuning	21.29					26.90				
LwF	22.90					26.21				
EWC	21.19					26.63				
Buffer Size	0.2k	0.5k	1k	2k	5k	0.2k	0.5k	1k	2k	5k
A-GEM	12.62	17.28	15.36	15.22	13.28	15.30	15.45	14.86	15.80	17.76
ASER	22.34	23.54	23.45	27.14	30.22	24.48	25.86	25.65	23.43	25.72
GDumb	14.68	15.52	15.88	16.99	**36.00**	14.09	15.17	15.79	17.41	**40.21**
GSS	23.00	22.55	25.42	20.64	25.34	24.63	21.90	20.10	22.22	27.12
ER	25.01	28.59	24.64	27.21	25.03	25.49	24.79	24.74	25.81	29.99
MIR	27.63	29.74	27.65	29.37	30.50	27.99	26.66	29.86	28.98	26.84
SCR	**27.77**	**30.99**	**33.26**	**32.39**	34.97	**28.00**	**31.66**	**30.85**	**34.57**	34.99

**Table 5:** Average accuracy (%) of the CL methods in UrbanSound8K in the ODI scenario. A higher score is better.

Dataset	UrbanSound8K									
ODI Scenario	Noise					SpecAugment				
Offline	33.24					34.15				
Online	27.89					25.40				
Fine-tuning	24.54					26.95				
LwF	26.21					28.53				
EWC	27.05					24.36				
Buffer Size	0.2k	0.5k	1k	2k	5k	0.2k	0.5k	1k	2k	5k
A-GEM	16.15	15.56	17.23	17.59	15.34	14.08	16.52	16.00	14.68	16.44
ASER	25.34	27.00	27.82	28.54	27.67	25.56	24.71	27.24	29.16	23.02
GDumb	17.53	16.28	14.37	30.08	33.63	15.86	16.83	14.79	29.56	34.19
GSS	24.05	22.82	21.89	27.11	24.56	23.34	23.64	24.37	28.61	29.87
ER	22.71	27.47	27.08	26.82	28.07	25.42	26.21	27.10	27.10	30.30
MIR	25.68	26.98	26.18	30.61	28.56	24.84	28.52	27.29	25.73	24.97
SCR	**33.82**	**36.51**	**35.37**	**35.73**	**35.60**	**34.25**	**35.36**	**35.30**	**35.10**	**36.60**

**Table 6:** Average forgetting (%) of CL methods in DCASE-2021 in the ODI scenario. A lower score is better.

Dataset	DCASE-2021									
Type of ODI	Noise					SpecAugment				
Fine-tuning	7.43					5.35				
LwF	5.44					2.46				
EWC	6.34					4.00				
Buffer Size	0.2k	0.5k	1k	2k	5k	0.2k	0.5k	1k	2k	5k
A-GEM	3.06	3.81	2.96	4.44	4.78	5.17	4.30	3.08	5.68	3.75
ASER	9.28	5.70	4.05	4.79	3.33	5.38	4.39	5.57	7.68	5.95
GDumb	1.21	2.37	1.50	3.53	1.06	2.70	1.89	2.61	2.51	1.86
GSS	1.28	2.37	1.77	3.03	3.23	**0.74**	**0.99**	**1.28**	0.97	2.27
ER	2.28	1.35	2.89	3.43	4.13	2.37	2.94	5.29	2.90	1.65
MIR	**1.14**	1.16	1.92	1.74	1.94	2.09	1.92	1.37	2.14	4.43
SCR	1.17	**1.08**	**0.53**	**0.52**	**0.55**	1.45	1.30	1.61	**0.34**	**0.32**

**Table 7:** Average forgetting (%) of CL methods in UrbanSound8K in the ODI scenario. A lower score is better.

Dataset	UrbanSound8K									
Type of ODI	Noise					SpecAugment				
Fine-tuning	7.60					5.06				
LwF	3.79					3.14				
EWC	4.44					5.82				
Buffer Size	0.2k	0.5k	1k	2k	5k	0.2k	0.5k	1k	2k	5k
A-GEM	5.73	4.55	2.90	3.09	4.21	5.27	6.01	3.69	3.71	3.59
ASER	5.90	6.02	6.64	5.04	4.69	6.87	5.63	7.63	4.47	8.12
GDumb	1.89	4.31	5.84	1.73	2.03	4.31	3.47	5.17	2.21	2.08
GSS	2.80	1.47	4.22	4.27	5.13	2.20	1.37	1.53	2.84	2.41
ER	5.27	2.76	4.56	4.18	2.90	2.66	5.07	5.43	5.43	2.92
MIR	4.03	3.44	3.95	**0.88**	3.66	5.06	1.25	3.98	3.66	7.05
SCR	**1.31**	**0.55**	**0.63**	**0.88**	**0.80**	**2.06**	**0.73**	**1.67**	**0.68**	**0.47**

## Data Availability

Data sharing not applicable.

## Abbreviations

The following abbreviations are used in this manuscript:

ASC	Acoustic scene classification
ASER	Adversarial Shapley value experience replay
ASV	Adversarial Shapley value
A-GEM	Averaged gradient episodic memory
CL	Continual learning
DCASE	The detection and classification of acoustic scenes and events
ER	Experience replay
EWC	Elastic weight consolidation
GDumb	Greedy sampler and dumb learner
GEM	Gradient episodic memory
GSS	Gradient-based sample selection
HAR	Human activity recognition
iCaRL	Incremental classifier and representation learning
ID	Identifier
LwF	Learning without forgetting
MIR	Maximally interfered retrieval
NCM	Nearest-class-mean
NLP	Natural language processing
OCI	Online class incremental
ODI	Online domain incremental
SCR	Supervised contrastive replay
SV	Shapley value
